# REV-ERB-Mediated Regulation of Neural Functional Mechanisms by Phytochemicals in the Circadian System

**DOI:** 10.3390/nu18183071

**Published:** 2026-09-20

**Authors:** Ming-Ying Pan, Jiang-Bao Wang, Run Li, Zi-Ying Wang, Mu-Wen Lu, Jun-Qing Huang

**Affiliations:** 1 Guangdong Provincial Key Laboratory of Nutraceuticals and Functional Foods, College of Food Science, South China Agricultural University, Guangzhou 510642, China; 13612453249@163.com; 2 School of Traditional Chinese Medicine, Jinan University, Guangzhou 510632, China; 3 Department of Food Science, Rutgers University, New Brunswick, NJ 08901, USA; run.li@rutgers.edu

**Keywords:** REV-ERB, circadian clock, central nervous system, phytochemicals

## Abstract

As a nuclear receptor in the circadian regulatory network, REV-ERB maintains circadian rhythm stability and participates in central nervous system (CNS) homeostasis by regulating clock-gene expression, emerging as a promising intervention target for neurodegenerative disorders. Beyond circadian regulation, it is indispensable for diverse critical physiological processes, including organ function modulation, metabolism, immune responses, and neural activity and transcriptional regulation. Many phytochemicals have been confirmed to regulate CNS function and systemic metabolism via REV-ERB and its downstream cascades. In this review, the regulatory potential of REV-ERB in CNS homeostasis and function is summarized. The roles of various phytochemicals in this regulatory pathway are examined, with a particular emphasis on their capacity to modulate neurological health. This work could enhance our understanding of REV-ERB-mediated neuroprotective and metabolic actions of phytochemicals, and provide a scientific basis for circadian-guided functional food design and the application of phytochemicals in preventing neurodegenerative diseases.

## 1. Introduction

Circadian rhythm is a universal biological system found in organisms ranging from animals to plants, fungi, and bacteria, regulating a wide range of physiological processes [1]. It is driven by an autonomous molecular oscillator, which operates through a self-regulating cycle of expression, accumulation, and degradation of clock gene products [2]. Although the central clock is located in the brain’s suprachiasmatic nucleus (SCN), circadian mechanisms are present in numerous cell types across the body, functioning autonomously from the central clockwork [3].

REV-ERBs, members of the nuclear receptor family, consist of two homologous proteins, REV-ERBα and REV-ERBβ, which are essential components of the regulatory network in the circadian system, maintaining clock rhythmicity and period stability [4,5].

Moreover, REV-ERB is involved in the expression of other core circadian clock genes, including *Bmal1*, *Clock*, and *RORs* [1]. In biological clock regulation, the BMAL1-CLOCK heterodimer binds to E-box elements in the promoters of downstream clock genes, including *Per1*, *Per2, Cry1*, *Cry2*, *Rev-erbα*, and *Rev-erbβ*, thereby initiating their transcription. PER and CRY proteins accumulate and subsequently repress the transcriptional activity of the BMAL1-CLOCK complex, forming the core negative feedback loop; *RORs* compete with *Rev-erb* to bind to the *Bmal1* promoter and activate its expression, maintaining the clock [6].

Ligands targeting REV-ERB have received much attention as potential therapeutics due to the critical role of REV-ERB in a variety of physiological processes. As a transcriptional repressor, REV-ERB ligands can be classified as agonists or antagonists based on their mechanism of action, while agonists can bind to REV-ERB and activate its function, and antagonists can inhibit the function of REV-ERB. The endogenous ligand heme also regulates REV-ERB by enhancing its inhibitory effect on target genes, thus influencing cellular metabolism and circadian rhythms [7].

**Figure 1:**
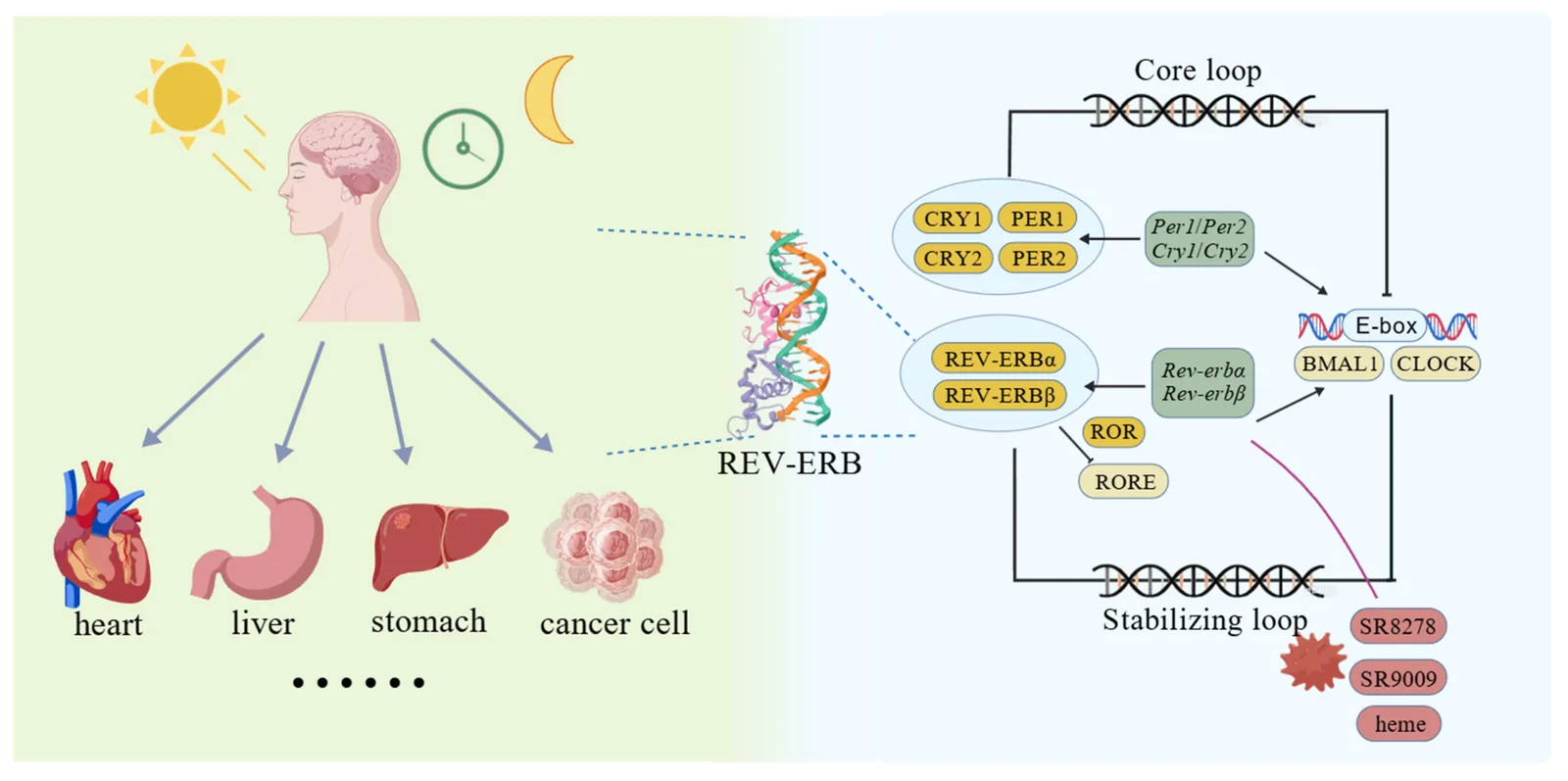
Schematic representation of the mammalian circadian clock regulatory network and the central role of REV-ERB in orchestrating systemic metabolism and central nervous system homeostasis.

REV-ERB also serves as a key target across various diseases and is essential for proper physiological functioning, including heart, metabolism, and immune response, and the regulation of neuroinflammation and circadian rhythms in the central nervous system [8]. The core mammalian circadian clock comprises the BMAL1/CLOCK-driven feedback loops and the competitive binding of REV-ERB and ROR, through which REV-ERB orchestrates systemic metabolism and central nervous system function across multiple organs (as shown in Figure 1). Phytochemicals are widely present in natural plant sources such as citrus peel, turmeric, and tea, and have recently attracted considerable attention due to their pleiotropic bioactivities and emerging roles as circadian clock modulators [9]. Accumulating evidence has demonstrated that various phytochemicals can regulate the rhythmic expression of core clock genes (such as *Bmal1*, *Rev-erbα*, and *Clock*), thereby exerting neuroprotective and metabolic regulatory effects [10,11]. However, despite the increasing evidence supporting the neuroprotective and metabolic regulatory effects of phytochemicals, their precise molecular mechanisms within the circadian system remain incompletely understood [12]. In particular, direct evidence demonstrating the interaction between phytochemicals and REV-ERB signaling is still limited [13]. Therefore, further studies are required to clarify whether phytochemicals regulate central nervous system function through REV-ERB-dependent pathways. Based on recent studies on the regulation of REV-ERB by phytochemicals, this review summarizes the regulatory roles of REV-ERB in the central nervous system and discusses the modulatory effects of several phytochemicals with significant neuroprotective and metabolic regulatory activities on REV-ERB, as well as their underlying molecular mechanisms. In this review, we distinguish direct evidence for REV-ERB-mediated effects, such as REV-ERB binding, transcriptional repression, or genetic requirement, from indirect evidence, such as circadian clock gene modulation without causal proof of REV-ERB engagement. This distinction is maintained throughout the review to avoid overinterpretation of correlational findings. This work could enhance our understanding of the REV-ERB-mediated neuroprotective and metabolic actions of phytochemicals, and provide a scientific basis for circadian-guided functional food design and their application in preventing neurodegenerative diseases. However, it should be noted that the effective doses used in preclinical studies are often not directly achievable through dietary intake, a limitation that will be further discussed at the end of Section 3.

## 2. The Central Regulatory Potential of REV-ERB

### 2.1. Neuroinflammation

Neuroinflammation refers to the inflammatory response occurring within the CNS, which is linked to disorders like stroke, Alzheimer’s disease, Huntington’s disease, and depression [14]. REV-ERBα has been reported to play a significant role in regulating neuroinflammation by modulating glial cell responses and immune signaling pathways [15,16]. Also, REV-ERBα influences the balance between pro- and anti-inflammatory mediators, thereby shaping the overall neuroinflammatory environment. Through these mechanisms, REV-ERBα not only regulates immune cell activity but also helps maintain a neuroprotective balance in the CNS [15].

According to Guo et al., REV-ERBα agonists inhibit LPS-induced phosphorylation of IκBα kinase (IκK); this inhibition prevents IκBα degradation and nuclear factor kappa B (NF-κB) nuclear translocation, thereby suppressing the expression and secretion of pro-inflammatory genes, including *IL-6* and *TNFα* [17]. Morioka et al. reported that the REV-ERBα agonist SR9009 suppresses the upregulation of Iba1 induced by LPS or partial sciatic nerve ligation (PSNL), and also reduces LPS-induced increases in *IL-1β* and *IL-6* mRNA in rat spinal microglial cells [18]. Mohammed Amir et al. reported that REV-ERBα regulates Th17 cells by inhibiting IL-17a expression through competition with *RORγt* for binding to DNA response elements, which downregulates other Th17-associated cytokines including IL-17F, IL-21, IL-22, IL-6 and TNFα [19].

Therefore, REV-ERBα has the potential to be used as an anti-neuroinflammatory agent by inhibiting the production of inflammatory factors through blocking NF-κB activation, modulating microglia, and antagonizing the Th17 pathway, thus suppressing neuroinflammation.

### 2.2. Neuroplasticity

Neuroplasticity serves as a foundational framework for understanding brain development, learning processes, and homeostatic regulation within the CNS [20]. It affects the synaptic phagocytosis of neuronal cells by modulating multiple molecular and cellular mechanisms associated with neuroplasticity, thereby impacting the structure and function of the brain [21]. REV-ERBα has been demonstrated to be involved in the regulation of neuroplasticity, thus influencing synaptic remodeling and neuronal function [22].

Sarrazin et al. reported that knocking down *Bmal1* in excitatory neurons of the medial prefrontal cortex (mPFC) in the mouse model not only blocked the antidepressant effect of ketamine but also downregulated the expression of Homer1a, a key molecule in synaptic plasticity [23,24]. Treatment with the REV-ERB agonist SR10067 downregulated both *Bmal1* and Homer1a in the mPFC, impairing the antidepressant efficacy of ketamine. Griffin et al. demonstrated that *Bmal1* deletion upregulated the expression of complement C4B (C4b) and Complement 3 (C3) in the hippocampus, which was further enhanced following *Rev-erb*α deletion, leading to enhanced synaptic phagocytosis by microglial cells, particularly in the CA3 region. Synaptic phagocytosis exhibited a circadian rhythm (peaking at ZT17; ZT refers to Zeitgeber time, with ZT0 indicating lights-on and ZT12 indicating lights-off) but was abolished in *Rev-erb*α-deficient mice, which displayed persistently elevated phagocytosis levels [22]. The proliferation and differentiation of adult hippocampal neural stem/progenitor cells (AHPs), key components of neuroplasticity, are regulated by dynamic molecular mechanisms [25]. Koji Shimozaki et al. reported that the REV-ERB agonist SR9009 exerted bidirectional effects on AHP proliferation and neurogenesis at different concentrations. At 0.1 µM, SR9009 promoted synaptic growth, while higher concentrations (2.5 µM) inhibited this process [26].

By modulating the *Bmal1*-Homer1a axis, adjusting the circadian rhythm of microglial synaptic phagocytosis, and influencing the proliferation and differentiation of AHPs, REV-ERB contributed to the maintenance of neuroplasticity, highlighting its role in managing synaptic homeostasis across neurodevelopmental and degenerative diseases.

### 2.3. Microglial Function

Microglia in the CNS function as the primary innate immune cells and the first responders to pathological insults [27]. These cells exhibit dual roles in pro-inflammatory or neuroprotective functions, categorized into two phenotypes based on their activation state: M1 (classical activation) and M2 (alternative activation) [28]. REV-ERBα has been shown to regulate microglial activation and metabolic processes [15,29].

According to Percy Griffin et al., REV-ERBα bound to the promoter regions of genes in the NF-κB signaling pathway, such as *Traf2* and *Nfκb2*, inhibiting their expression and thereby maintaining microglia in a resting state [15]. In the absence of REV-ERBα, *Traf2* expression was upregulated, NF-κB signaling was enhanced, and this promoted pro-inflammatory cytokine (such as IL-1β, TNFα, IL-6) expression [15]. Samantha et al. reported that REV-ERBα agonist SR9011 reduced microglial cellular energy metabolism by inhibiting the mitochondrial respiratory chain, reducing oxygen consumption and adenosine triphosphate (ATP) production. SR9011 downregulated the expression of key metabolic genes, including *carnitine palmitoyltransferase 1* (*Cpt1*), *pyruvate dehydrogenase kinase 1* (*Pdk1*), and *hexokinase 2* (*Hk2*), which are involved in important cellular metabolic pathways such as fatty acid oxidation, glycolysis, and the citric acid cycle [29].

In summary, REV-ERBα modulated microglial immune responses and energy metabolism by regulating NF-κB signaling genes and mitochondrial activity, processes that contributed to the pathogenesis of neurological disorders.

### 2.4. Amyloid Plaque Deposition

The defining pathological hallmarks of Alzheimer’s disease (AD) are the presence of extracellular amyloid-beta (Aβ) plaques -aggregates of Aβ peptides-and neurofibrillary tangles composed of hyperphosphorylated tau protein filaments [30]. The inheritance of the APOE4 allele exacerbates AD progression by inhibiting Aβ clearance from the brain interstitial fluid, enhancing microglial activation, and promoting proinflammatory cytokine production [31]. Emerging evidence suggests that REV-ERB may alleviate AD pathology by regulating Aβ metabolism [32].

According to Lee et al., knocking out the *Rev-erbα* gene in animal models reduced the number and size of amyloid plaques in 5XFAD mice and decreased the expression of disease-related microglial markers, including *TREM2*, *CD45*, and *Clec7a* [32,33]. In vitro, inhibition of REV-ERBs enhanced microglial uptake of Aβ, aligning with the peak expression timing of the circadian clock gene *Bmal1*. Marottoli et al. found that REV-ERB agonist SR9009 reduced mitochondrial superoxide levels and cell stiffness in APOE4 brain endothelial cells while decreasing chemokine levels, such as MCP-1/CCL2 and KC/CXCL1 [34].

Therefore, REV-ERB reduced Aβ peptide accumulation in AD by regulating amyloid plaque number and size, enhancing microglial Aβ clearance, and modulating ApoE expression, thereby alleviating Alzheimer’s pathology.

### 2.5. Neurometabolism

Neurometabolic abnormalities may function not only as chronic concomitants of epilepsy, but also as direct factors in epileptogenesis [35]. Consequently, modulating neurometabolic pathways has emerged as a viable strategy for effective seizure control. Emerging evidence further indicates that circadian clock genes extend beyond their canonical role in the maintenance of circadian rhythms, actively participating in the regulation of neurometabolic processes [36].

Regulation of neurometabolism includes pathways such as the synthesis of gamma-aminobutyric acid (GABA) and the mammalian target of rapamycin (mTOR) signaling pathway [37]. Accumulating research evidence demonstrates pyridoxine metabolism is essential for the biosynthesis of the neurotransmitter GABA, a process intricately associated with epileptogenesis [38]. Although few studies have directly established a link between REV-ERB and the regulation of enzymes in pyridoxal metabolism, evidence exists that these enzymes are under circadian control [39]. Dadon- Amador et al. found that REV-ERBα activated the mTOR complex 1 (mTORC1) signaling pathway by inhibiting the transcription of tuberous sclerosis complex 1 (Tsc1), an endogenous inhibitor of mTORC1, and this action also affected the phosphorylation level of BMAL1, thereby inducing alterations in circadian rhythms [40,41].

Therefore, REV-ERB regulated neurometabolism by modulating the circadian rhythm of enzymes involved in pyridoxine metabolism and by activating the mTORC1 signaling pathway through the inhibition of Tsc1 transcription.

### 2.6. Sleep and Appetite Regulation

Research has demonstrated that REV-ERB modulates sleep-associated and orexigenic genes [42]. Its mechanism of action involves the transcriptional control of core circadian clock genes. Disruptions in circadian rhythms are often associated with sleep and appetite disorders, in which REV-ERB, a key regulator of the biological clock, exerts a regulatory effect [43].

According to Amador et al., *Rev-erbβ* knockout mice exhibited significantly reduced wakefulness during the dark phase, while their slow-wave sleep and rapid eye movement sleep increased. Besides, the absence of REV-ERBβ led to the upregulation of sleep-promoting genes, such as *fos proto-oncogene* (*Fos*), *gamma-aminobutyric acid type A receptor subunit alpha5* (*Gabrα5*), and wake-promoting genes, such as *histamine receptor 1* (*Hrh1*), revealing the mechanisms by which REV-ERBβ regulated the balance between sleep-wake balance [44].

Orexins, neuropeptides produced in the hypothalamus, contribute to the regulation of sleep/wake cycles through complex interactions with monoaminergic, cholinergic, and GABA-ergic neuronal systems [45]. Feillet et al. reported that *Rev-erbα* knockout mice exhibited increased intake and preference for high-reward foods, including such as sucrose and chocolate, with this phenotype being more pronounced during the nocturnal active phase, and in hypothalamus, REV-ERBα repressed the expression of the orexin by binding to the ROR elements (RORE) in the orexin promoter, thereby regulating appetite [46].

In summary, REV-ERB regulated sleep and appetite by modulating the expression of sleep- associated genes such as *Fos* and *Gabra5* and by influencing the expression of orexin. These findings indicate that REV-ERBα plays an important role in coordinating sleep–wake behavior and metabolic processes, further highlighting its broader regulatory functions beyond the core circadian clock.

### 2.7. C/EBP Family-Associated Transcriptional Regulation

Accumulating evidence suggests that REV-ERBα may participate in broader transcriptional regulation beyond the core circadian clock, including cellular stress, inflammation, and neuronal function [47]. The CCAAT/enhancer-binding protein (C/EBP) family, including C/EBPα, C/EBPβ, and C/EBP homologous protein (CHOP), has been implicated in neuronal development, neuroinflammation, and neurodegenerative diseases [48,49]. In particular, C/EBPα has been implicated in neuronal development and AD-related pathology, with recent evidence showing that C/EBPα transactivates MIR128-1 and regulates miR-128 expression, thereby influencing tau phosphorylation and amyloid-β accumulation [50].

Zhou et al. reported that REV-ERBα activated C/EBP homologous protein (CHOP, DDIT3) transcription through a RORE-containing region in the Chop promoter, providing direct evidence for transcriptional regulation of a C/EBP family member by REV-ERBα [51]. Furthermore, Shim et al. identified a REV-ERBα–C/EBPβ–SAA1 axis, showing that REV-ERBα activation impaired C/EBPβ binding to the SAA1 promoter and suppressed SAA1-associated inflammatory responses and pyroptosis [52]. More recently, Wang et al. demonstrated that C/EBPβ regulates delta-secretase expression and promotes amyloid and Tau pathology, neuroinflammation, and cognitive impairment in AD models [49].

Together, these findings support a functional connection between REV-ERBα and C/EBP family-mediated transcriptional regulation in inflammatory and neurodegenerative processes. However, whether REV-ERBα directly regulates C/EBPα or C/EBPβ in CNS-resident cells remains to be determined.

**Figure 2:**
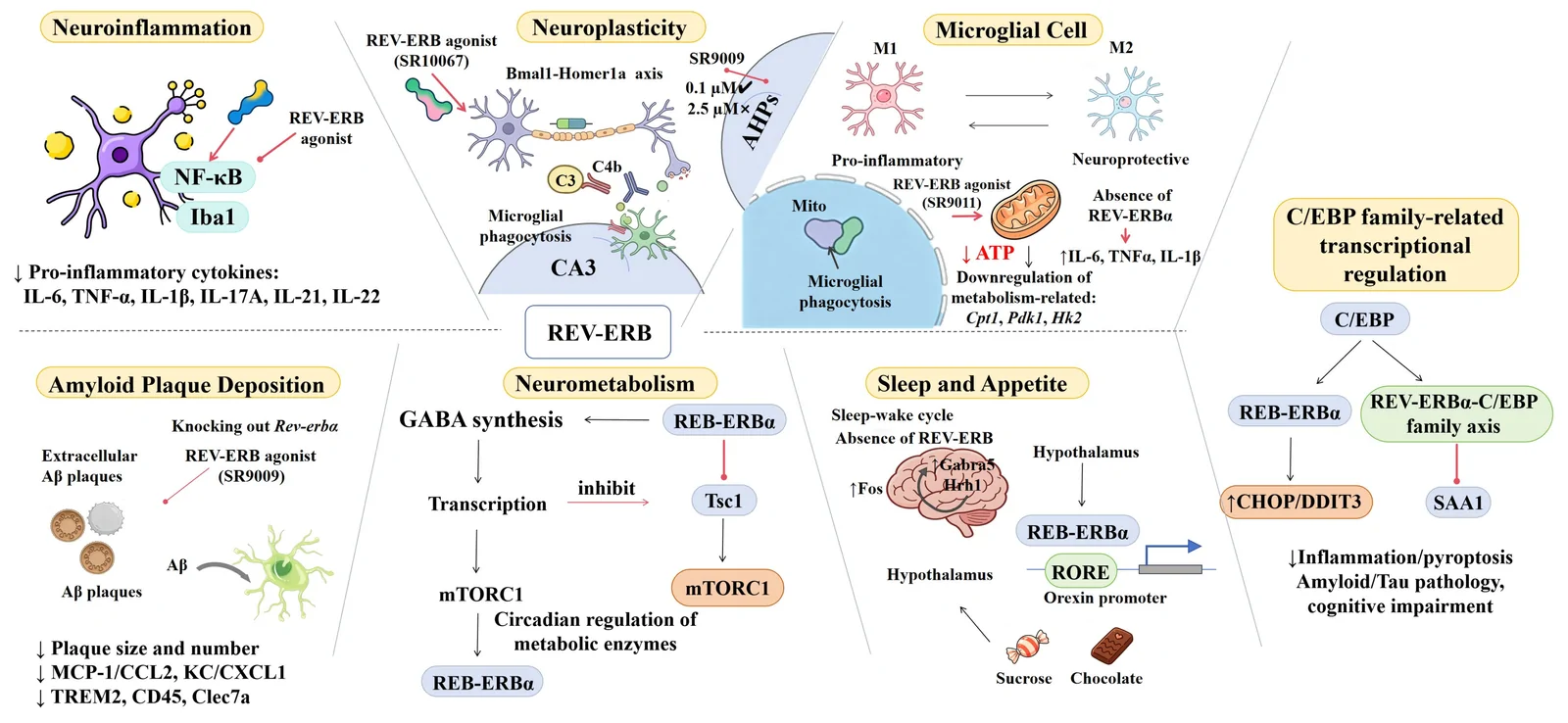
REV-ERB integrates circadian signals to regulate neuroinflammation, synaptic plasticity, microglial function, Aβ clearance, sleep/appetite, and C/EBP family-associated transcriptional regulation, thereby linking circadian regulation to neuroinflammatory and neurodegenerative processes. The pathways illustrated are compiled from studies on neuroinflammation [13,15,17], neuroplasticity [20,22,24], microglial function [13,27], Aβ clearance [30,32], sleep/appetite regulation [40,42,44], and C/EBP family-associated transcriptional regulation [50,51,52]. Note: ↑, increased or upregulated; ↓, decreased or downregulated.

Collectively, these findings highlight the multifaceted regulatory functions of REV-ERBα in the CNS, extending from circadian rhythm regulation and neuroinflammation to neuroplasticity and C/EBP family-associated transcriptional regulation, and provide a mechanistic basis for its therapeutic potential, as summarized in Table 1 and Figure 2.

## 3. Phytochemicals Regulate Nervous and Metabolic Systems Through REV-ERB in the Circadian Clock

Having established the regulatory roles of REV-ERB in various CNS functions in Section 2, we now turn to examine how phytochemicals may modulate these processes through REV-ERB-related pathways. This section is organized by individual phytochemicals, each discussed with respect to its effects on circadian clock genes, neurological outcomes, and systemic metabolism. The evidence presented here ranges from direct REV-ERB targeting, as demonstrated for puerarin, to indirect circadian clock modulation, as observed for EGCG and proanthocyanidins. This distinction is indicated in Table 2.

### 3.1. Nobiletin

**Figure 3:**
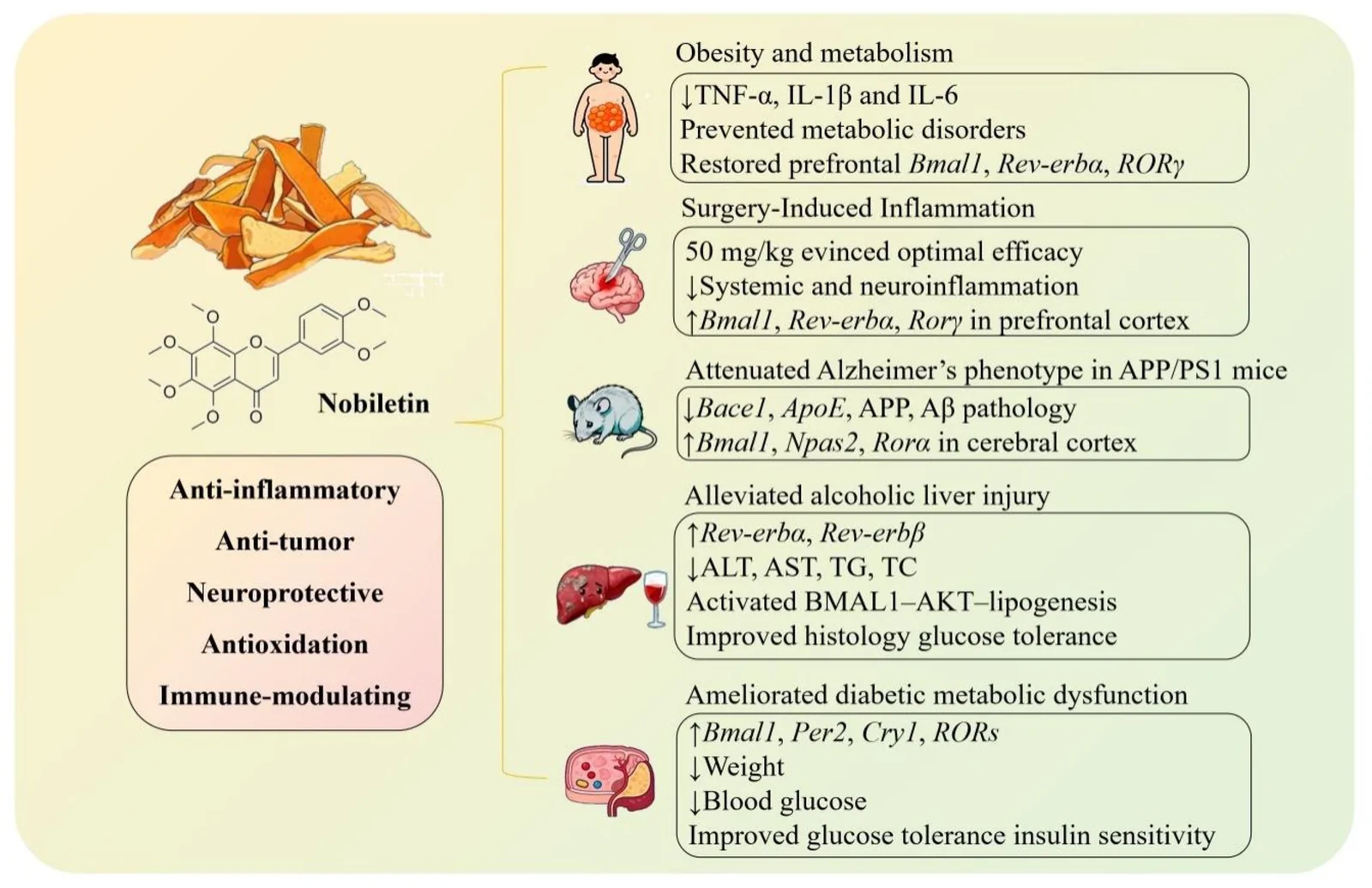
Regulatory effects and underlying molecular mechanisms of citrus peel-derived nobiletin (NOB) on systemic metabolism and neurological function via targeting REV-ERB and other canonical circadian clock genes to ameliorate circadian disruption-related neurodegenerative and metabolic disorders. The pathways illustrated are compiled from studies on NOB regulation of circadian clock genes and neuroinflammation [53], suppression of pro-inflammatory factors [53], attenuation of Aβ pathology [11], regulation of lipid metabolism [77], and activation of RORs and clock gene proteins [78].

Nobiletin (NOB), a polymethoxyflavonoid derived from peels, exhibits diverse bioactivities, including anti-inflammatory, anti-tumor, and neuroprotective effects, as well as the ability to mitigate oxidative stress and modulate immune responses [77,78]. Previous studies demonstrated that NOB prevented metabolic disorders associated with obesity by enhancing circadian rhythms in a manner dependent on circadian-related genes such as *Bmal1* [79]. Sun et al. demonstrated that pretreatment with NOB could dose-dependently inhibit both systemic and neuroinflammation induced by surgery—with the 50 mg/kg dose showing particular efficacy [54]. They further revealed that NOB reversed surgery-induced reductions in the expression of circadian clock genes (including *Bmal1*, *Rev-erbα*, and *Rorγ*) in the prefrontal cortex of mice, while simultaneously suppressing the production of pro-inflammatory factors such as TNF-α, IL-1β, and IL-6. According to Kim et al., NOB decreased the expression of AD-related genes *Bace1* and *ApoE* in the cerebral cortex of amyloid precursor protein/presenilin 1 (APP/PS1) double-transgenic mice, reduced APP protein levels, and improved Aβ pathology [11]. It also activated the expression of core clock genes in the cerebral cortex, including *Bmal1*, *Npas2* and *Rorα*, potentially influencing circadian rhythm regulation and thus improving AD pathology. According to Li et al., NOB restored the alcohol-induced downregulation of *Rev-erα* and *Rev-erβ* expression and reduced alcohol-induced elevations in ALT, AST, TG, and TC levels, while improving liver pathology and glucose tolerance by activating the BMAL1–AKT–lipogenesis pathway, thereby alleviating alcoholic liver disease [55]. He et al. found that NOB regulated the balance between RORs and REV-ERBs by activating RORs and increasing the protein levels of clock genes such as *Bmal1*, *Per2*, and *Cry1* [56]. These actions led to reductions in weight gain, lower blood glucose levels, and improvements in glucose tolerance and insulin sensitivity in diabetic mice, thereby effectively improving metabolic function (as shown in Figure 3).

Therefore, NOB regulated the circadian rhythm genes (such as *Bmal1* and *Rev-erbα*), suppressed the expression of AD-related genes (such as *Bace1* and *ApoE*) and pro-inflammatory factors, and reduced the levels of ALT, AST, TG, and TC, thereby exerting beneficial effects against neuroinflammation, Aβ pathology, alcohol-induced liver injury, and metabolic disorders.

### 3.2. Curcumin

Curcumin, a bioactive compound derived from the rhizome of turmeric, exhibits a wide range of pharmacological properties, including antioxidant, anti-inflammatory, anti-tumor, and lipid-lowering activities in vivo [57,58]. Aging erodes circadian signaling and clock-protein levels, undermining cellular chemistry and metabolic balance [59]. Kukkemane et al. revealed that curcumin supplementation restored the *Per1*, *Per2*, *Cry1*, and *Cry2* phases in middle-aged (12 months) rats and the *Per1* phase and daily pulse in aged (24 months) rats [10]. They also found that curcumin increased the expression levels of *Bmal1*, *Per2*, *Cry1*, and *Cry2* in the SCN. According to Thummadi et al., curcumin was found to restore the expression of clock genes in the kidneys of aging rats and reduce the expression of immune genes, including *Nfκb1*, *TNFα*, *IL-6*, *Tlr4* and *Tlr9* [60]. Sarma et al. reported that in rat C6 glioma cells, 5 μM curcumin induced rhythmic apoptosis peaking 6–11 h before *Per2* maximum, whereas 10 μM curcumin suppressed mitosis and disrupted the circadian regulation of cell death [61]. Of note, such micromolar concentrations of curcumin are rarely attainable in human plasma after oral administration due to its extremely low bioavailability and extensive first-pass metabolism; its safety profile, including the LD_50_, has been documented in toxicological studies [62].

In summary, curcumin supplementation restored aging-disrupted circadian rhythms and clock gene expression, including *Bmal1*, *Per2*, *Cry1*, and *Cry2* in SCN, as well as *Rev-erbα* in kidneys. It also attenuated the expression of immune-related genes and exerted central protective effects by interacting with the circadian system in a dose-dependent manner.

### 3.3. (-)-Epigallocatechin-3-gallate

The green tea polyphenol (-)-epigallocatechin-3-gallate (EGCG), the most abundant catechin and a major active component in green tea, has been shown to ameliorate diet-induced metabolic disturbances by regulating the rhythmic expression of clock genes in multiple tissues, such as the liver, adipose tissue, and the intestine, and additionally exerts antioxidant, anti-carcinogenic, anti-inflammatory, and cardioprotective effects [63,80,81].

**Figure 4:**
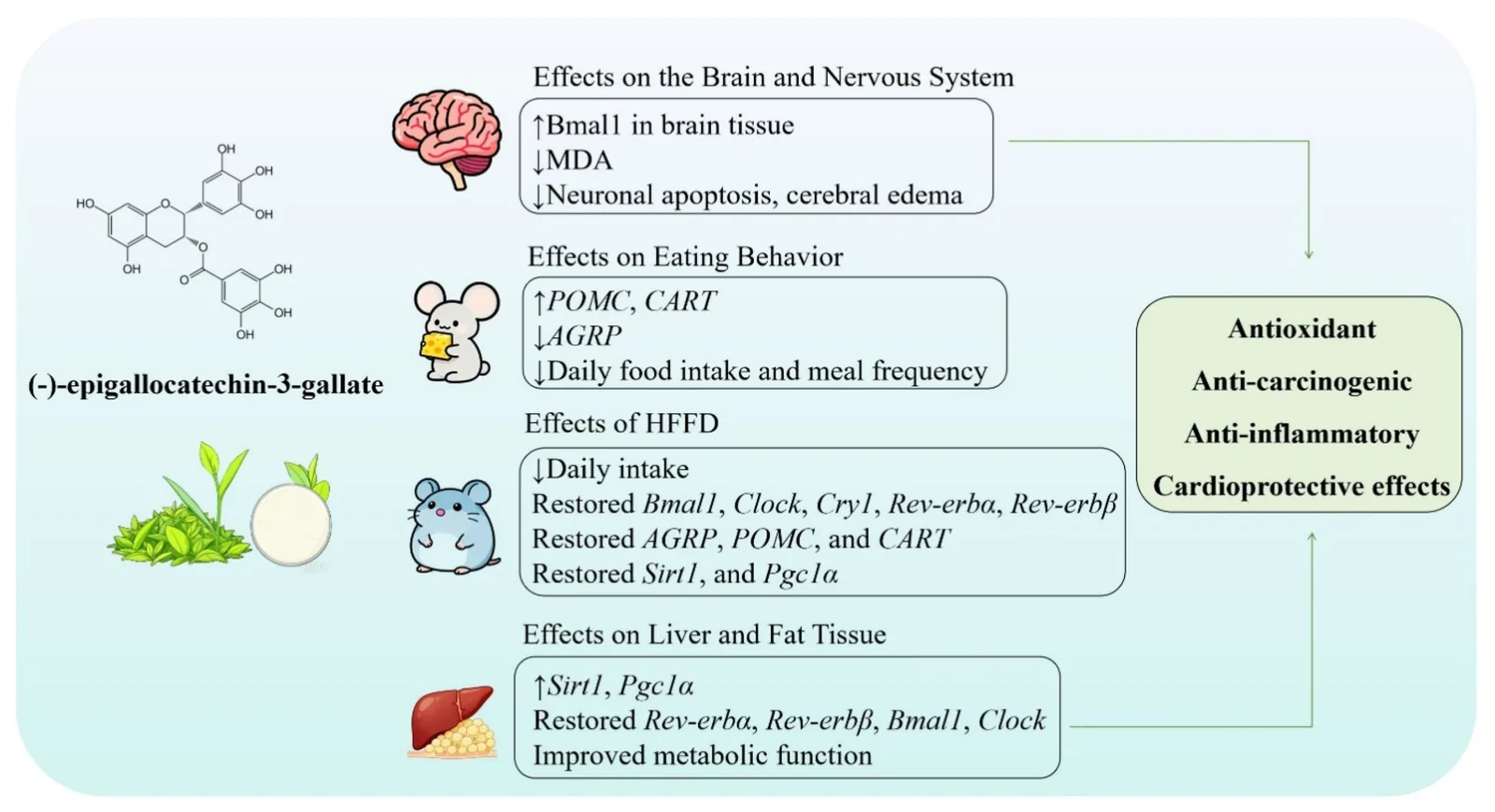
Regulatory effects and underlying molecular mechanisms of green tea-derived epigallocatechin gallate (EGCG) on systemic metabolism and neurological function via targeting REV-ERB and other canonical circadian clock genes to ameliorate circadian disruption-related neurodegenerative and metabolic disorders. The pathways illustrated are compiled from studies on ECGG regulation of appetite genes and eating behavior [60], elevation of *Bmal1* and reduction of oxidative stress [62], and restoration of clock genes in hypothalamus, liver, and adipose tissue [63,80].

Li et al. reported that EGCG increased circadian rhythm genes [63]. They further discovered that EGCG could regulate the eating behavior of obese mice induced by a high-fat diet by affecting the expression of appetite-regulating genes, including decreased *agouti-related peptide* (*AGRP*), *pro-opiomelanocortin* (*POMC*), and *cocaine-amphetamine-regulated transcript* (*CART*) in the hypothalamus; this, in turn, reduced daily food intake amount and frequency. Yu et al. found that EGCG (100 mg/kg, intraperitoneal injection) in a rat surgical brain injury model elevated *Bmal1* expression in brain tissue and reduced the oxidative stress marker malondialdehyde (MDA) [64]. The results also showed that EGCG attenuated neuronal apoptosis and degeneration and alleviated cerebral edema. According to Mi et al., EGCG could restore the disrupted rhythmic expression of clock genes (such as *Bmal1*, *Clock* and *Cry1*) and appetite regulation genes (such as *AGRP*, *POMC* and *CART*) in the hypothalamus induced by high-fat and high-fructose diet (HFFD) through enhancing their circadian rhythm amplitude, thereby improving metabolic disorders and eating behavior [65]. Mi et al. also discovered that EGCG improved the rhythmic disruption of circadian rhythm genes, including *Bmal1*, *Clock*, *Rev-erbα*, and *Rev-erbβ*, in liver and adipose tissue caused by HFFD [66]. It further affected the expression of *sirtuin 1* (*Sirt1*) and *peroxisome proliferator-activated receptor coactivator 1-α* (*Pgc1α*), thereby improving metabolic function, as depicted in Figure 4.

Therefore, EGCG restored the amplitude and phase of circadian rhythm genes (such as *Bmal1*, *Clock*, *Rev-erbα*) and appetite-regulating genes (such as *AGRP*, *POMC*, and *CART*) in the hypothalamus, liver, and fat, thereby improving metabolic disorders with a protective effect on the central nervous system.

### 3.4. Puerarin

As the key isoflavone extracted from Pueraria lobata, puerarin possesses vasodilatory, anti-inflammatory, anti-apoptotic, and hypoglycemic properties [82]. It exerts neuroprotective and cardioprotective effects and is widely used as an adjuvant in the clinical management of cardiovascular diseases [83,84]. Recent evidence indicated that puerarin alleviated hyperhomocysteinemia in mice through *Rev-erbα* targeting [67]. According to Chen et al., puerarin upregulated the expression of the key homocysteine-metabolizing enzymes betaine homocysteine methyltransferase (BHMT), cystathionine β-synthase (CBS), and cystathionine γ-lyase (CTH) by blocking the transcriptional repression mediated by *Rev-erbα* [67]. This regulatory effect was abolished in *Rev-erbα*-deficient cells, which indicated that puerarin exerted its protective effect against hyperhomocysteinemia by antagonizing the transcriptional repression mediated by *Rev-erbα*. Liu et al. demonstrated that puerarin targeted *Rev-erbα* in the DSS-induced chronic colitis model, with administration at ZT10 more effectively suppressing the colonic expression of inflammatory factors such as *NOD-like receptor family pyrin domain containing* 3 (*Nlrp3*), *IL-1β*, *IL-6*, *TNFα*, and *C-C motif chemokine ligand 2* (*Ccl2*) than at ZT2, and alleviated colon injury [68].

Therefore, puerarin targeted *Rev-erbα* to derepress the homocysteine-clearing enzymes BHMT, CBS, and CTH, lowered homocysteine, and in a time-dependent manner suppressed colonic *Nlrp3*, *IL-1β*, *IL-6*, *TNF-α*, and *Ccl2*, thereby exerting vasodilatory, anti-inflammatory, and cardio-neuroprotective effects.

### 3.5. Capsaicin

**Figure 5:**
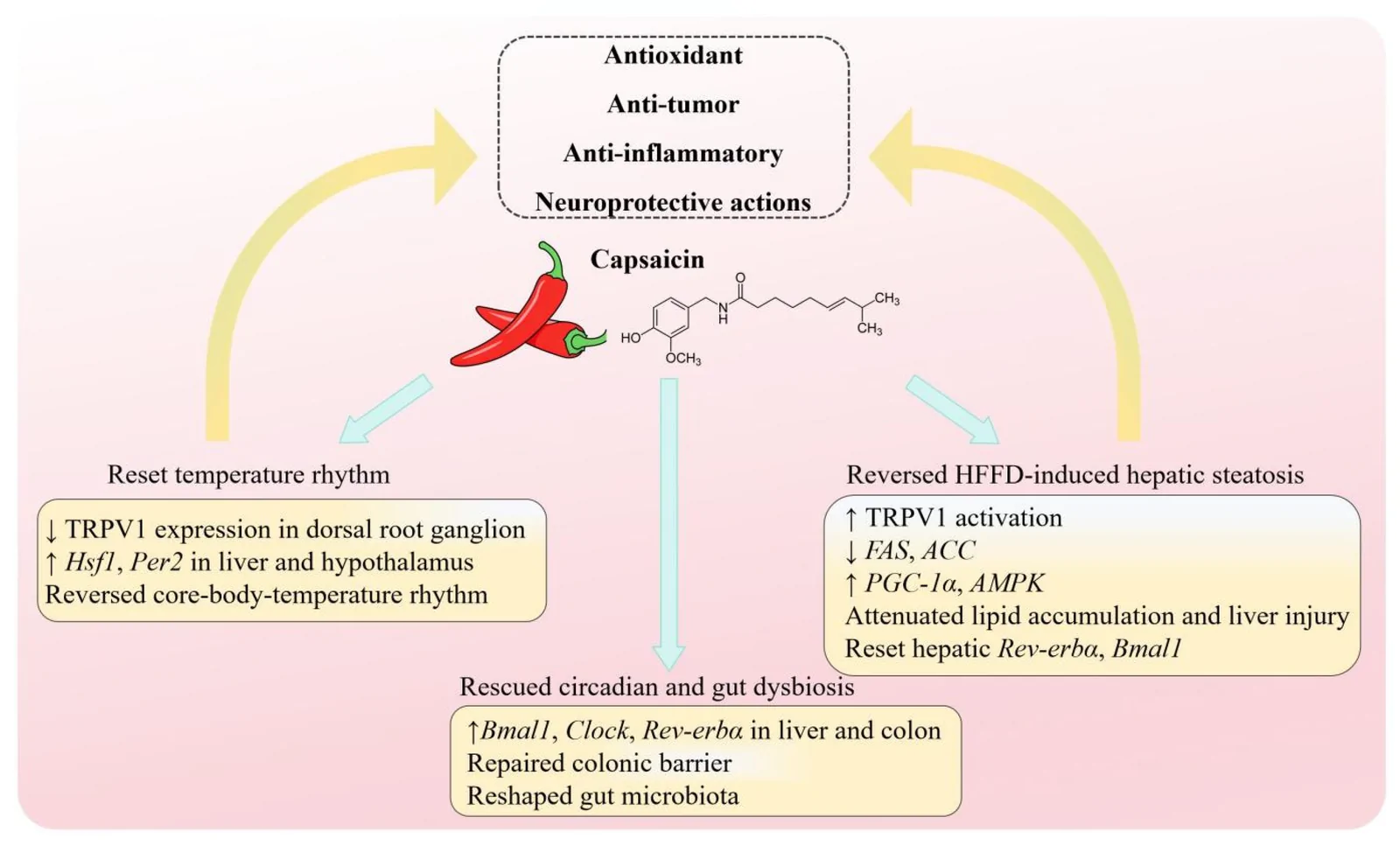
Regulatory effects and underlying molecular mechanisms of chili pepper-derived capsaicin (CAP) on systemic metabolism and neurological function via targeting REV-ERB and other canonical circadian clock genes to ameliorate circadian disruption-related neurodegenerative and metabolic disorders. The pathways illustrated are compiled from studies on CAP modulation of circadian clock genes and metabolism [67], TRPV1-related neural temperature rhythm [68], restoration of liver and colon clock gene expression [48,85], and REV-ERBα-mediated lipid metabolism via a TRPV1-dependent mechanism [48].

Capsaicin (CAP) is the active ingredient in chili pepper, which has antioxidant, anti-tumor, anti-inflammatory, and neuroprotective effects [85]. CAP can be used to relieve pain, lose weight, and protect against cardiovascular and cerebrovascular diseases, and it shows prospects in neuroprotective, anti-inflammatory, and cognitive and emotional improvement [86]. Recent studies have shown that CAP could regulate the expression of biological clock genes, thereby affecting metabolism [87]. Jeong et al. discovered that injection of CAP (50 mg/kg) during the neonatal period reduced transient receptor potential vanilloid 1 (TRPV1) expression in the dorsal root ganglion of Sprague-Dawley (SD) rats and impaired their perception of harmful heat [69]. They further revealed that the circadian core body temperature rhythm in the CAP-treated rats was reversed, and this disruption was associated with abnormally elevated hepatic and hypothalamic expression of *heat shock factor 1* (*Hsf1*) and *Per2*. According to Deng et al., in the mouse model of circadian rhythm disorder induced by continuous darkness, CAP repaired the colonic barrier and reshaped the diversity of the intestinal flora [70]. At the same time, CAP restored the expression of circadian rhythm genes (such as *Bmal1*, *Clock*, and *Rev-erbα*) in the liver and colon. Cao et al. reported that CAP activated the TRPV1 channel and restored the circadian disruption of hepatic clock genes (such as *Rev-erbα*, *Bmal1*) induced by HFFD [71]. Furthermore, they found in HepG2 cells that *Rev-erbα* knockdown abolished CAP-induced suppression of *fatty acid synthase* (*FAS*), *acetyl-CoA carboxylase* (*ACC*), and restoration of *AMPK* and *Pgc-1α*, mirroring TRPV1 knockdown. This indicates CAP regulates REV-ERBα-mediated lipid metabolism in a TRPV1-dependent manner, providing direct cellular evidence that REV-ERBα is a key downstream target of CAP’s metabolic regulation, thereby alleviating hepatic lipid accumulation and injury, as presented in Figure 5.

In summary, CAP activated TRPV1 channels, modulated core clock gene expression in the liver, colon, and hypothalamus, and restored circadian rhythms disrupted by a high-fat diet or constant darkness, thereby ameliorating liver injury and intestinal dysbiosis while also affecting the neural perception of noxious heat.

### 3.6. Resveratrol

**Figure 6:**
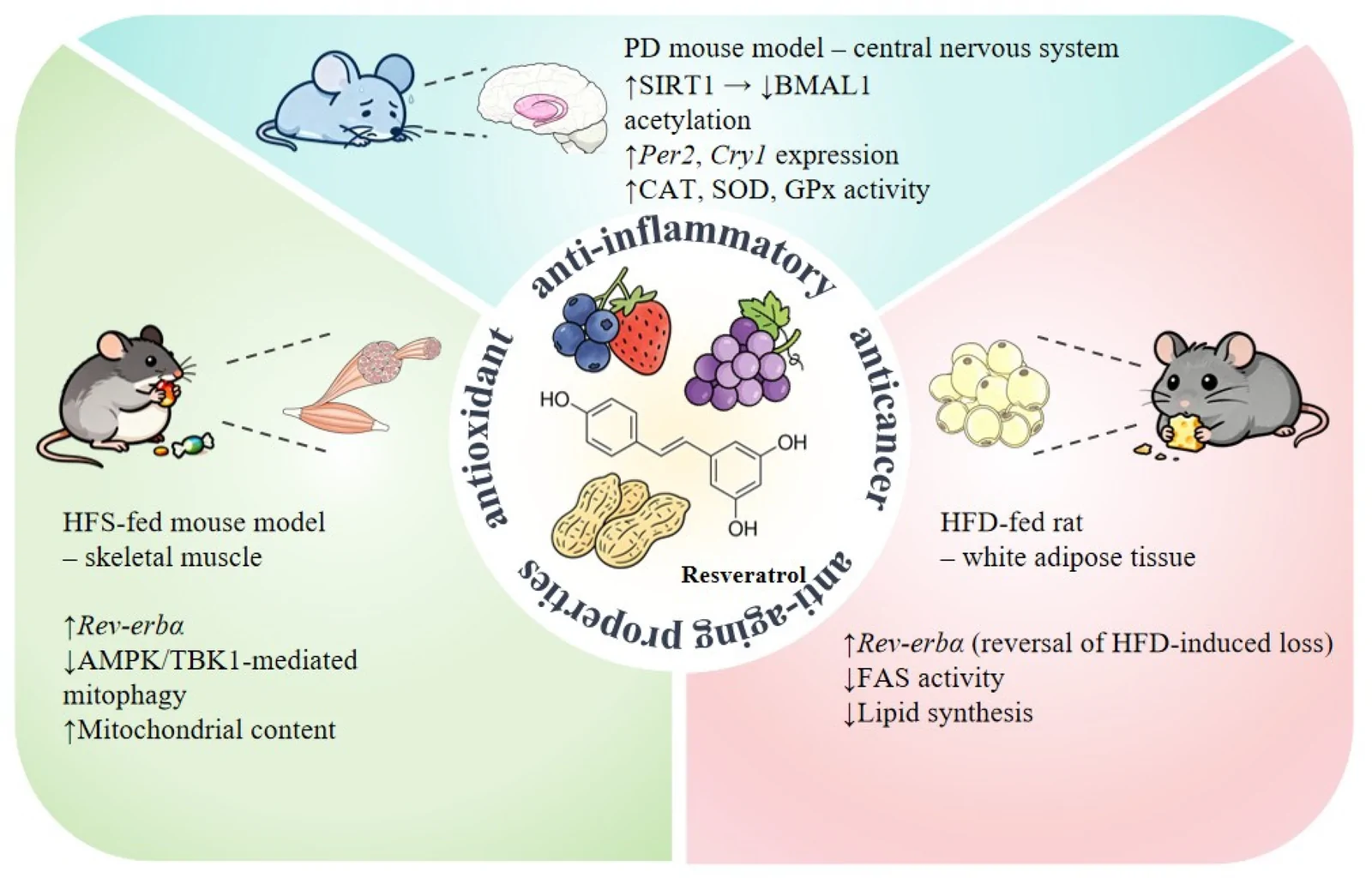
Regulatory effects and underlying molecular mechanisms of grape- and berry-derived resveratrol on systemic metabolism and neurological function via targeting REV-ERB and other canonical circadian clock genes to ameliorate circadian disruption-related neurodegenerative and metabolic disorders. The pathways illustrated are compiled from studies on resveratrol activation of SIRT1 and restoration of clock genes in a PD model [70], upregulation of *Rev-erbα* and inhibition of AMPK/TBK1 mitophagy [71], and reversal of aberrant *Rev-erbα* expression and lipid metabolism in adipose tissue [88].

Resveratrol is a natural polyphenolic compound found in foods such as grapes, peanuts, and berries, known for its antioxidant, anti-inflammatory, anticancer, and anti-aging properties [88,89]. Recent studies have revealed that resveratrol also showed potential in regulating circadian rhythms [90]. According to Wang et al., in a Parkinson’s disease (PD) model, resveratrol reduced BMAL1 acetylation levels in the striatum of the central nervous system by activating SIRT1, thereby restoring the expression of circadian rhythm genes (such as *Per2* and *Cry1*) and enhancing the activity of antioxidant enzymes, including catalase (CAT), superoxide dismutase (SOD), and glutathione peroxidase (GPx) in dopaminergic neurons [72]. Hyatt et al. reported that resveratrol upregulated *Rev-erbα* expression and suppressed the AMPK/TANK-binding kinase 1 (TBK1)-mediated mitophagy pathway, which attenuated the high-fat/high-sucrose (HFS)-induced decline in skeletal-muscle mitochondrial content [73]. Miranda et al. found that resveratrol reversed the HFD-induced aberrant expression of *Rev-erbα* in rat white adipose tissue and was accompanied by reduced FAS activity and lipid synthesis [74]. (as shown in Figure 6).

In summary, resveratrol improved antioxidant capacity in the PD model by activating SIRT1 and reducing BMAL1 acetylation; it also inhibited AMPK/TBK1-mediated mitophagy while elevating *Rev-erbα* to reverse diet-induced metabolic decline in muscle and fat.

### 3.7. Proanthocyanidins

Proanthocyanidins (PAs), also known as condensed tannins, are flavonoid polymers that play a role in determining the flavor and astringency of various beverages such as teas, wines, and juices [91]. Studies have been conducted on their potential health benefits, including their impact on metabolism and circadian rhythm regulation [92]. According to Ribas-Latre et al., PAs administered during the ZT0 modulated the expression of the hypothalamic *Bmal1* and downstream target nicotinamide phosphoribosyltransferase (NAMPT), thereby altering plasma melatonin levels and the circadian oscillation of multiple metabolites [75]. When PAs were given at ZT12, they up-regulated the mRNA level of *Rev-erbα*, suggesting that *Rev-erbα* represented a time-sensitive regulator in the clock network. Ribas-Latre et al. also reported that PAs up-regulated NAMPT expression, increased nicotinamide adenine dinucleotide (NAD^+^) levels, and enhanced BMAL1 acetylation in both healthy and obese rats [76]. This mechanism further modulated the expression of circadian clock genes, including *Bmal1*, *Clock*, *Per2*, *Rev-erbα*, and *Rorα*, in the liver, intestine, and white adipose tissue.

In summary, proanthocyanidins exerted time-dependent control of circadian clock genes—suppressing *Bmal1*/NAMPT at ZT0 and inducing *Rev-erbα* at ZT12—and alleviated obesity-related circadian and metabolic disruption by activating the NAMPT–NAD^+^ pathway in the liver, intestine, and adipose tissue (as shown in Table 2). It should be noted that the effective doses and concentrations used in the cited animal and cell studies are often considerably higher than those achievable through dietary intake of the corresponding plant sources. For instance, the 50 mg/kg dose of nobiletin used in mice would require an adult human to consume approximately 4–5 g of pure compound daily, equivalent to nearly 5 kg of fresh citrus peels; curcumin at 10 µM is rarely attainable in human plasma after oral administration due to its extremely low bioavailability; and the plasma EGCG levels from drinking green tea are in the nanomolar to low-micromolar range, far below the concentrations frequently used in mechanistic studies [93,94,95]. Similarly, challenges such as the poor oral bioavailability of puerarin and resveratrol, as well as the limited absorption of proanthocyanidins, further underscore the gap between experimental pharmacology and practical dietary outcomes [96,97]. Therefore, while these phytochemicals demonstrate clear REV-ERB-modulating activities and serve as valuable lead compounds, their documented pharmacological effects should not be directly equated with the benefits of consuming the corresponding whole foods. Nevertheless, the development of purified extracts, advanced formulations, or structurally optimized derivatives holds promise for translating these mechanistic insights into practical applications.

## 4. Discussion and Perspectives

As a nuclear receptor within the circadian regulatory network, REV-ERB participates in CNS homeostasis by governing clock-gene expression and has emerged as a promising intervention target in neurodegenerative disorders. Phytochemicals such as NOB, Curcumin, EGCG, and other natural active ingredients have been reported to affect central nervous system function and body metabolism by regulating REV-ERB and its downstream pathways.

Beyond summarizing existing findings, this review argues that REV-ERB deserves special attention as a key hub that connects metabolism, immunity, and circadian regulation within the CNS. Unlike *Per* and *Cry*, which mainly function as feedback repressors of transcription, REV-ERB directly represses metabolic and inflammatory genes such as *NF-κB* and *Nlps3*. This property allows REV-ERB to bridge circadian disruption and downstream neuropathological events [15,17]. Importantly, the emerging connection between REV-ERBα and the C/EBP family further expands its regulatory network beyond canonical circadian pathways. These findings suggest a potential link between REV-ERB-associated transcriptional regulation, inflammation, and neurodegenerative processes [50,51,52]. However, whether these regulatory relationships directly operate in CNS-resident cells remains to be established. Therefore, targeting REV-ERB with phytochemicals represents a promising strategy for intervening in the core pathogenic mechanisms shared across neurodegenerative diseases. Synthetic REV-ERB agonists, such as SR9009 and SR9011, and antagonists, such as SR8278, have been instrumental in elucidating these functions, yet many exhibit limited isoform selectivity and off-target effects [98,99]. Compared with these synthetic ligands, phytochemicals generally show weaker potency but offer favorable safety profiles and structural diversity, serving as valuable lead compounds for developing next-generation REV-ERB modulators [100].

Among the reviewed compounds, nobiletin and resveratrol have been reported to exert direct actions within the CNS. Nobiletin suppresses neuroinflammation in the prefrontal cortex and attenuates Aβ pathology, while resveratrol restores clock gene expression in the striatum through the SIRT1-BMAL1 pathway [11,54,72]. These findings support a direct CNS model of action. For compounds such as EGCG and capsaicin, although the current evidence does not establish a causal link between peripheral REV-ERB regulation and central outcomes, their potential to influence CNS function through indirect pathways should not be overlooked. Both compounds have been shown to modulate REV-ERB rhythmicity in peripheral tissues including the liver, adipose tissue, and gut, while separately exhibiting neuroprotective effects [65,66,70,87]. Importantly, the route from peripheral metabolic regulation to central nervous system modulation is biologically plausible. It is well established that gut microbiota-derived metabolites, particularly short-chain fatty acids (SCFAs), can cross the blood-brain barrier and modulate brain functions via G protein-coupled receptors, histone deacetylase inhibition, and neuroimmune pathways [101,102,103]. SCFAs have been shown to regulate microglial function, synaptic plasticity, and blood-brain barrier integrity, and their production is itself influenced by circadian rhythms [104,105]. Thus, phytochemicals that modulate REV-ERB in peripheral tissues could theoretically influence CNS function through such metabolic intermediaries. In addition to microbiota-derived metabolites, circulating inflammatory mediators may represent another important pathway linking peripheral tissues and the CNS. Peripheral immune activation can communicate with the brain through circulating cytokines, including IL-6 and TNF-α, thereby influencing neuroimmune responses through blood-borne signaling pathways [106,107]. Peripheral REV-ERB modulation induced by phytochemicals may potentially affect systemic inflammatory profiles. Therefore, peripheral metabolic and immune signals may serve as potential communication routes between peripheral tissues and the central nervous system [108]. However, direct experimental evidence confirming these mechanisms remains limited and requires further investigation [109]. However, direct experimental evidence connecting phytochemical-induced peripheral REV-ERB modulation to central outcomes via these pathways remains scarce, representing an important direction for future investigation.

In addition, although this review focuses on seven representative phytochemicals with relatively well-characterized REV-ERB-related activities, we acknowledge that other classes of natural compounds may also hold promise. Carotenoids, stilbenes such as pterostilbene, and curcumin derivatives have been reported to modulate core clock gene expression or ROR-mediated transcriptional pathways [10,110,111]. However, direct experimental evidence for their interaction with REV-ERB is still lacking, and most reported effects are confined to peripheral tissues, leaving their CNS efficacy largely unexplored. These compounds represent attractive candidates for future screening, but their neuroprotective relevance through REV-ERB should be interpreted with caution at this stage.

Although previous studies have revealed the multiple functions of REV-ERB in the central nervous system, the current research on the regulation of nerve function by phytochemicals through REV-ERB is still in its preliminary stage, and there are many unresolved problems. First, most existing evidence centers on REV-ERBα, whereas its homolog REV-ERBβ remains underexplored. The scarcity of REV-ERBβ studies is largely due to the lack of isoform-selective pharmacological tools and its later discovery [100,112]. REV-ERBβ plays unique roles in neuronal differentiation and wakefulness, and recent studies indicate that the two isoforms can compensate for each other in the brain—dual deletion unmasks changes in protein catabolism, complement, and oxidative stress that are not seen with REV-ERBα deletion alone [113]. Second, the neural system-specific research models remain imperfect; most studies focus on peripheral tissues, lacking functional verification systems based on neurons [114]. For example, Griffin et al. and Lee et al. constructed systemic *Rev-erbα* deletion models to evaluate microglial synaptic pruning and Aβ clearance, but brain region-specific knockout models are still lacking [22,32]. Third, beyond its role in Aβ metabolism, REV-ERB has also been implicated in tau and α-synuclein pathologies [113,115,116]. Microglial REV-ERBα deletion impairs tau phagocytosis and exacerbates tau aggregation, while its inhibition improves tauopathy [115,116]. Moreover, REV-ERBα and REV-ERBβ coordinately regulate astrocytic α-synuclein clearance, and their dual deletion mitigates α-synuclein spreading in Parkinson’s models [113]. REV-ERBα also attenuates NLRP3-mediated neuroinflammation in Parkinson’s disease [15].

In addition, the dose-response effects of phytochemicals, the duration of intervention, and their interaction with circadian phenotypes have not been systematically elucidated, limiting their practical application in functional foods and nutritional interventions [117]. For example, NOB restored surgery-induced clock gene disruption and suppressed neuroinflammation in a dose-dependent manner by targeting the REV-ERB pathway [54]. And ZT12 administration of PAs upregulated the expression of REV-ERB*α* in the brain, but ZT0 administration had no effect [75]. It is also important to acknowledge that the phytochemicals discussed in this review, including isoflavones such as puerarin and stilbenes such as resveratrol, are known to interact with multiple molecular targets beyond REV-ERB. For instance, resveratrol activates SIRT1, AMPK, and estrogen receptors, while puerarin modulates PI3K/Akt and NF-κB pathways [118,119,120]. Such pleiotropic activities, while contributing to their broad health benefits, complicate the attribution of observed neuroprotective effects specifically to REV-ERB modulation and raise concerns about target selectivity in vivo. Furthermore, safety considerations should not be overlooked. Although these compounds are generally well tolerated at dietary levels, high-dose supplementation or pharmacological use may pose risks. Polyphenols, including resveratrol and EGCG, have been associated with pro-oxidant effects, hepatotoxicity, and gastrointestinal intolerance at elevated doses, and green tea extract has been linked to liver injury in susceptible individuals [121,122]. These findings underscore the need for rigorous dose-response characterization and long-term safety evaluation in future translational studies. Future studies should systematically compare different doses, intervention timing, and inter-individual circadian variation to define an individualized dosing window for phytochemicals.

While this review highlights the emerging roles of phytochemicals in REV-ERB-mediated neural regulation, it also underscores that rigorous mechanistic validation and chronobiology-guided study designs remain urgently needed to translate these findings into clinical applications. Therefore, the specific mechanisms of phytochemicals regulating neural function through the REV-ERB pathway in humans, the dose effects, and their interactions with individual circadian phenotypes need to be systematically studied, and future research should be strengthened in the following directions. To explore the synergistic effects of different phytochemicals through REV-ERB at the cell, tissue, and system levels, especially in neuroprotection and metabolic regulation. In view of the differences in absorption, distribution, metabolism, and excretion of phytochemicals in the body, it is necessary to strengthen the study of their pharmacokinetic properties, especially the activity and tissue distribution of metabolites. Many phytochemicals have problems such as low bioavailability and poor penetration of the blood-brain barrier. In the future, new delivery systems (such as nanocarriers, liposomes, targeted modification molecules) should be developed to achieve central targeted delivery at lower doses and reduce the risk of potential toxicity. This review is helpful in improving our understanding of the neuroprotective and metabolic actions of phytochemicals mediated by REV-ERB, and in providing a scientific basis for circadian-guided functional food design and for their application against neurodegenerative diseases.

## Figures and Tables

**Table 1:** Mechanisms of REV-ERB regulation in the central nervous system.

Neurological Process	Location	REV-ERB Transcriptional Repressor Activity	Effects and Mechanisms	Ref.
Neuroinflammation	Microglia, spinal cord, Th17	↑ REV-ERBα	↓ Phosphorylation of the IκBα kinase; ↓ Degradation of IκBα; ↓ The nuclear translocation of NF-κB; ↓ RORγt; ↑ Iba1; ↓ IL-6, TNF-α, IL-1β, IL-17A, IL-21, IL-22;	[17,18,19]
Neuroplasticity	mPFC, hippocampus, AHP	↑ REV-ERBα	↓ *Bmal1*, Homer1a; ↓ The antidepressant effect of ketamine; ↑ Synaptic growth (0.1 µM, SR9009); ↓ Synaptic growth (2.5 µM, SR9009)	[24,26]
	CA3 region	↓ REV-ERBα	↑ C3, C4b ↑ Synaptic phagocytosis;	[22]
Microglial function	Cultured microglia, CNS	↑ REV-ERBα	↓ *Traf2*, *Nfkb2*, NF-κB; ↓ Mitochondrial respiratory chain; ↓ Oxygen consumption and ATP production; ↓ *Cpt1*, *Pdk1*, *Hk2*;	[29,53]
	CNS	↓ REV-ERBα	↑ IL-1β, TNF-α, IL-6;	[53]
Amyloid plaque deposition	Brain endothelial cells	↑ REV-ERBα	↓ Mitochondrial superoxide levels, cell stiffness; ↓ MCP-1/CCL2, KC/CXCL1;	[33]
	Hippocampus, cerebral cortex	↓ REV-ERBα	↓ Aβ plaque size/number, ↓ *TREM2*, *CD45*, *Clec7a*; ↑ Microglial uptake of Aβ;	[34]
Sleep and appetite	CNS	↓ REV-ERBβ	*↑ Fos*, *Gabra5*, *Hrh1*;	[44]
	Hypothalamus	↓ REV-ERBα	↓ *Orexin*;	[46]
C/EBP family-related transcriptional regulation	Myeloid cells	↑ REV-ERBα	↑ CHOP/DDIT3;	[51]
		REV-ERBα-C/EBP family axis	↓ C/EBPβ binding to *Saa1* promoter; ↓ SAA1 transcription; ↓ Pyroptosis	[52]

Note: ↑, increased or upregulated; ↓, decreased or downregulated.

**Table 2:** Regulating effects of phytochemicals on REV-ERB and other circadian clock genes in neurological and metabolic functions.

Phytochemicals	Key Food Source	Direct Regulation	Central Clock Regulation	Peripheral Clock Regulation	Clock Target	Neural Effects	Metabolic Effects	Ref.
Nobiletin	Citrus peel	Not investigated	Yes (restored the alcohol-induced downregulation of REV-ERBα/β)	Yes (Reverses Surgery-Induced REV-ERBα Reduction)	BMAL1, REV-ERBα, REV-ERBβ, ROR, PER2, CRY1	Dose-dependently inhibited both systemic and neuroinflammation induced by surgery; mitigated Aβ burden via down-regulation of APP, BACE1, and APOE	↓ Alcohol-elevated ALT/AST/TG/TC; ↑ BMAL1–AKT-lipogenesis; Improved liver and glucose tolerance; ↓ Weight, blood glucose, insulin resistance	[11,54,55,56]
Curcumin	Turmeric (rhizome)	Not investigated	Not Investigated	Yes (Restore REV-ERBα expression in kidney)	BMAL1, REV-ERBα, PER1, PER2, CRY1, CRY2	Restored SCN clock gene rhythms; induced rhythmic apoptosis in C6 glioma cells.	↓ Expression of immune genes (*Nfκb1*, *TNF-α*, IL-6, *Tlr4*, *Tlr9*)	[10,57,58,59,60,61,62]
EGCG	Green tea	Not investigated	Not Investigated	Yes (Hepatic/Adipose REV-ERBα/β Rhythmic Restoration)	BMAL1, CLOCK, REV-ERBα, REV-ERBβ, CRY1	Reduced oxidative stress and neuronal apoptosis; improved brain injury-induced neurological deficits	Regulated appetite genes (AGRP, POMC, CART); Improved metabolic rhythm in liver and adipose tissue.	[63,64,65,66]
Puerarin	Pueraria lobata	Yes	Not Investigated	Yes (REV-ERBα Repression Antagonism)	REV-ERBα	Was not clearly stated	↑ Homocysteine-clearing enzymes (BHMT, CBS, CTH); ↓ Time-dependent colonic inflammatory cytokines	[67,68]
Capsaicin	Chili pepper	Yes	Not Investigated	Yes (Cellular Regulation via REV-ERBα Inhibition)	BMAL1, CLOCK, REV-ERBα, PER2	Restored TRPV1-related neural temperature rhythm abnormalities caused by circadian disruption	Repaired intestinal barrier; Reshaped gut microbiota; Restored hepatic clock gene expression and lipid metabolism	[69,70,71]
Resveratrol	Grapes, red wine, peanuts, berries	Not investigated	Not Investigated	Yes (REV-ERBα Upregulation)	BMAL1, CLOCK, REV-ERBα, PER2, CRY1	Reduced striatal acetylation; restored clock genes; boosted antioxidant enzymes in dopamine neurons	↓ AMPK/TBK1 mitophagy; Restored muscle mitochondria and adipose Rev-erbα/FAS/lipid balance	[72,73,74]
Proanthocyanidins	Teas, wines, and juices	Not investigated	Yes (Upregulates Hypothalamic Rev-erbα mRNA)	Yes (Upregulates Rev-erbα)	BMAL1, CLOCK, REV-ERBα, PER2, RORα	Modulated hypothalamic NAMPT; shifted plasma melatonin and metabolite rhythms	Time-dependently regulated clock genes; ↑ NAD^+^ levels; ↓ Obesity-related metabolic disruption	[75,76]

Note: ↑, increased or upregulated; ↓, decreased or downregulated.

## Data Availability

No new data were generated or analyzed in this review article. All information discussed in this study was obtained from previously published literature.

## Abbreviations

ACC, Acetyl-CoA carboxylase; AD, Alzheimer’s disease; AGRP, Agouti-related peptide; AHPs, Adult hippocampal neural stem/progenitor cells; ALT, Alanine aminotransferase; Ampk, AMP-activated protein kinase; APOE4, Apolipoprotein E4; AST, Aspartate aminotransferase; ATP, Adenosine triphosphate; Aβ, Amyloid-beta; BHMT, Betaine homocysteine methyltransferase; Bmal1, Brain and Muscle ARNT-Like 1; C3, Complement 3; C4b, Complement C4B; CAP, Capsaicin; CART, Cocaine-amphetamine-regulated transcript; CAT, Catalase; CBS, Cystathionine β-synthase; Ccl2, C-C motif chemokine ligand 2; CD45, C/EBP, CCAAT/enhancer-binding protein; CHOP, C/EBP homologous protein; Cluster of differentiation 45; Clec7a, C-type lectin domain family 7, member A; Clock, Circadian locomotor output cycles kaput; CNS, Central nervous system; Cpt1, Carnitine palmitoyltransferase 1; Cry1, Cryptochrome 1; Cry2, Cryptochrome 2; CTH, Cystathionine γ-lyase; DARPP-32, cAMP-regulated phosphoprotein of 32 kDa; DDIT3, DNA damage-inducible transcript 3; EGCG, (–)-epigallocatechin-3-gallate; FAS, Fatty acid synthase; Fos, Fos proto-oncogene; GABA, Gamma-aminobutyric acid; Gabrα5, Gamma-aminobutyric acid type A receptor subunit alpha5; GPx, Glutathione peroxidase; HD, Huntington’s disease; HFFD, High-fat and high-fructose diet; HFS, High-fat/high-sucrose diet; Hrh1, Histamine receptor 1; Hsf1, Heat shock factor 1; Hk2, Hexokinase 2; IκK, IκBα kinase; IL-1β, Interleukin-1 beta; IL-6, Interleukin 6; IL-17A, Interleukin-17A; IL-17F, Interleukin-17F; IL-21, Interleukin 21; IL-22, Interleukin 22; LPS, Lipopolysaccharide; MDA, Malondialdehyde; mPFC, Medial prefrontal cortex; mTOR, Mammalian target of rapamycin; mTORC1, Mechanistic Target of Rapamycin Complex 1; NAMPT, Nicotinamide phosphoribosyltransferase; NAD^+^, Nicotinamide adenine dinucleotide; NF-κB, Nuclear factor kappa B; Nfκb1, Nuclear factor kappa B subunit 1; Nfκb2, Nuclear factor kappa B subunit 2; NOB, Nobiletin; Npas2, Neuronal PAS domain protein 2; Nlrp3, NOD-like receptor family pyrin domain containing 3; PAs, Proanthocyanidins; Pdk1, Pyruvate dehydrogenase kinase 1; Per1, Period 1; Per2, Period 2; PGC-1α, Peroxisome proliferator-activated receptor coactivator 1-α; POMC, Pro-opiomelanocortin; Pparα, Peroxisome proliferator-activated receptor alpha; PSNL, Partial sciatic nerve ligation; REV-ERBα, Nuclear Receptor Subfamily 1 Group D Member 1 (NR1D1); REV-ERBβ, Nuclear Receptor Subfamily 1 Group D Member 2 (NR1D2); RORs, Retinoic Acid-Related Orphan Receptors; RORα, Retinoic Acid-Related Orphan Receptor Alpha; RORγt, Retinoid-related orphan receptor gamma t; RORE, Retinoic Acid-Related Orphan Receptor Response Element; SAA1, serum amyloid A1; SCN, Suprachiasmatic Nucleus; SD, Sprague-Dawley; Sirt1, Sirtuin 1; SOD, Superoxide dismutase; TBK1, TANK-binding kinase 1; TC, Total cholesterol; TG, Triglyceride; TNFα, Tumour necrosis factor α; Traf2, TNF receptor-associated factor 2; TREM2, Triggering receptor expressed on myeloid cells 2; TRPV1, Transient receptor potential vanilloid 1; Tlr4, Toll-like receptor 4; Tlr9, Toll-like receptor 9; Tsc1, Tuberous sclerosis complex 1.
